# External Validation of the Augmented Renal Clearance Predictor in Critically Ill COVID-19 Patients

**DOI:** 10.3390/antibiotics12040698

**Published:** 2023-04-03

**Authors:** Chao-Yuan Huang, Fabian Güiza, Matthias Gijsen, Isabel Spriet, Dieter Dauwe, Yves Debaveye, Marijke Peetermans, Joost Wauters, Greet Van den Berghe, Geert Meyfroidt, Greet De Vlieger

**Affiliations:** 1 Laboratory of Intensive Care Medicine, Academic Department of Cellular and Molecular Medicine, Katholieke Universiteit Leuven, 3000 Leuven, Belgium; 2 Department of Intensive Care Medicine, University Hospitals Leuven, 3000 Leuven, Belgium; 3 Pharmacy Department, University Hospitals Leuven, 3000 Leuven, Belgium; 4 Department of Pharmaceutical and Pharmacological Sciences, Katholieke Universiteit Leuven, 3000 Leuven, Belgium; 5 Laboratory for Clinical Infectious and Inflammatory Disorders, Department of Microbiology, Immunology and Transplantation, Katholieke Universiteit Leuven, 3000 Leuven, Belgium; 6 Medical Intensive Care Unit, Department of General Internal Medicine, University Hospitals Leuven, 3000 Leuven, Belgium

**Keywords:** renal elimination, COVID-19, intensive care units, machine learning, external validation, augmented renal clearance

## Abstract

The ARC predictor is a prediction model for augmented renal clearance (ARC) on the next intensive care unit (ICU) day that showed good performance in a general ICU setting. In this study, we performed a retrospective external validation of the ARC predictor in critically ill coronavirus disease 19 (COVID-19) patients admitted to the ICU of the University Hospitals Leuven from February 2020 to January 2021. All patient-days that had serum creatinine levels available and measured creatinine clearance on the next ICU day were enrolled. The performance of the ARC predictor was evaluated using discrimination, calibration, and decision curves. A total of 120 patients (1064 patient-days) were included, and ARC was found in 57 (47.5%) patients, corresponding to 246 (23.1%) patient-days. The ARC predictor demonstrated good discrimination and calibration (AUROC of 0.86, calibration slope of 1.18, and calibration-in-the-large of 0.14) and a wide clinical-usefulness range. At the default classification threshold of 20% in the original study, the sensitivity and specificity were 72% and 81%, respectively. The ARC predictor is able to accurately predict ARC in critically ill COVID-19 patients. These results support the potential of the ARC predictor to optimize renally cleared drug dosages in this specific ICU population. Investigation of dosing regimen improvement was not included in this study and remains a challenge for future studies.

## 1. Introduction

Augmented renal clearance (ARC) is common in critically ill patients, with a reported prevalence varying between 20 and 65% [1]. While there is no generally accepted ARC definition, it is commonly defined by creatinine clearance (CrCl) higher than 130 mL/min/1.73 m^2^ [1], measured within the past 8 to 24 h window depending on the urine collection time [2]. As the kidneys are an important route for drug elimination, ARC leads to decreased exposure to commonly used antibiotics such as beta-lactams and vancomycin [3,4,5] as well as anticoagulants [6]. Consequently, increased antibiotic doses are necessary for patients with ARC to increase exposure and decrease the risk of treatment failure [7].

Given the impact of ARC on renal drug clearance, many prediction models for ARC have been developed during the past few years [8,9,10]. The ARC score developed by Udy et al. is a point-based scoring system to predict ARC based on the adjusted odds ratios [8]. This model demonstrated decent predictive performance with an area under the receiving operating characteristics curve (AUROC) of 0.89 in a cohort of 71 critically ill sepsis and trauma patients. Additionally, the Augmented Renal Clearance of Trauma in Intensive Care (ARCTIC) score proposed by Barletta et al. is also point-based [9]. Recently externally validated [11], the ARCTIC score showed decent discrimination with an AUROC of 0.765 and 0.748 in the trauma and medical/surgical subgroups, respectively. Finally, the ARC predictor that was developed to predict the presence of ARC on the next intensive care unit (ICU) day outperformed the two existing models (ARC Score [8] and ARCTIC Score [9]) in the validation cohort [10] and has been made publicly available as an online calculator [12]. Although the ARC predictor showed promising results, its performance in other independent patient populations remains to be investigated before it can be recommended for broad clinical use [13].

Since the beginning of 2020, ICUs worldwide have been overwhelmed by a large number of critically ill coronavirus disease 19 (COVID-19) patients. More than 525 million people have been infected, and over 6 million people have died from COVID-19 [14]. Patients with COVID-19 mainly present with respiratory failure, but many critically ill COVID-19 patients also suffer from kidney dysfunction, with an acute kidney injury (AKI) prevalence of 18–81% [15]. On the other side of the renal function spectrum, ARC is common in critically ill COVID-19 patients, with a prevalence of 25–72% [16]. While the epidemiology of ARC has been described in critically ill COVID-19 patients [16,17,18,19,20], the performance of prediction models for ARC has not been reported in this patient population. Therefore, we aim to externally validate the ARC predictor in previously unseen critically ill COVID-19 patients.

## 2. Results

### 2.1. Study Cohort

**Figure 1:**
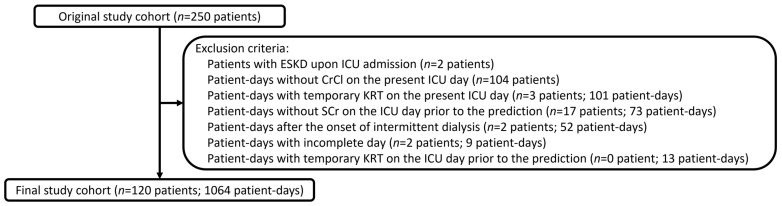
Consort diagram. ESKD, end-stage kidney disease; CrCl, creatinine clearance; KRT, kidney replacement therapy; SCr, serum creatinine; ICU, intensive care unit.

In total, 120 patients (1064 patient-days) were included, and ARC was found on at least one ICU day in 57 (47.5%) patients, corresponding to 246 patient-days (23.1%) (Figure 1). The descriptive statistics per patient and per patient-day are shown in Table 1 and Table S1 from supplementary. Seventy-two percent of the study cohort were male patients. Baseline serum creatinine (SCr) was missing in 178 (16.7%) patient-days, corresponding to 20 (16.7%) patients, and imputed with a median baseline SCr of 0.86 mg/dL. The median (interquartile range (IQR)) age was 67 (59–75) years, the median (IQR) body mass index (BMI) was 28.7 (25.8–33.1) kg/m^2^, and the median (IQR) ICU length of stay (LOS) was 14 (9–24) days. Patients with ARC were significantly younger (61 (57–67) vs. 73 (65–78) years, *p* < 0.01), had a lower baseline SCr (0.9 (0.7–0.9) vs. 0.9 (0.9–1.1), *p* < 0.01), and a lower Acute Physiology and Chronic Health Evaluation II (APACHE II) score (18 (13–23)) vs. 19 (17–27), *p* < 0.01). In ARC patients, the median (IQR) first day of ARC was day 1 (1–2) from ICU admission, the median (IQR) percentage of ARC days was 61.5% (25.0–100.0%), and the median (IQR) days with ARC was 2 (1–6) days (Figure S1 from supplementary). Patient-days with ARC had significantly higher CrCl (152.7 (138.3–175.6) vs. 74.8 (52.3–102.2) mL/min/1.73 m^2^, *p* < 0.01). In comparison with the original ARC predictor development cohort, this study cohort consisted of 10% more men (72.5 vs. 62.5%), had comparable ages (67 vs. 65 years), and showed an ICU LOS almost twice as long (14 vs. 8 days).

### 2.2. ARC Predictor External Validation Performance

**Figure 2:**
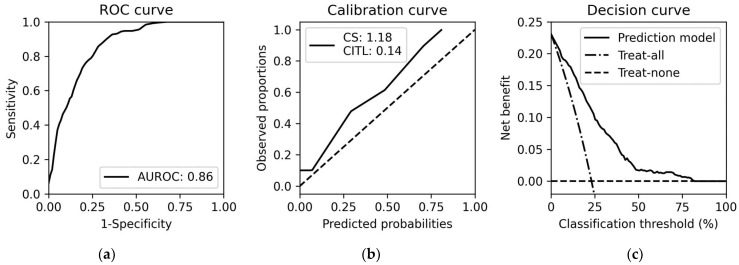
ARC predictor performance represented by (**a**) ROC curve; (**b**) calibration curve (**c**) decision curve. AUROC, area under the receiver operating characteristics curve; CS, calibration slope; CITL, calibration-in-the-large.

**Figure 3:**
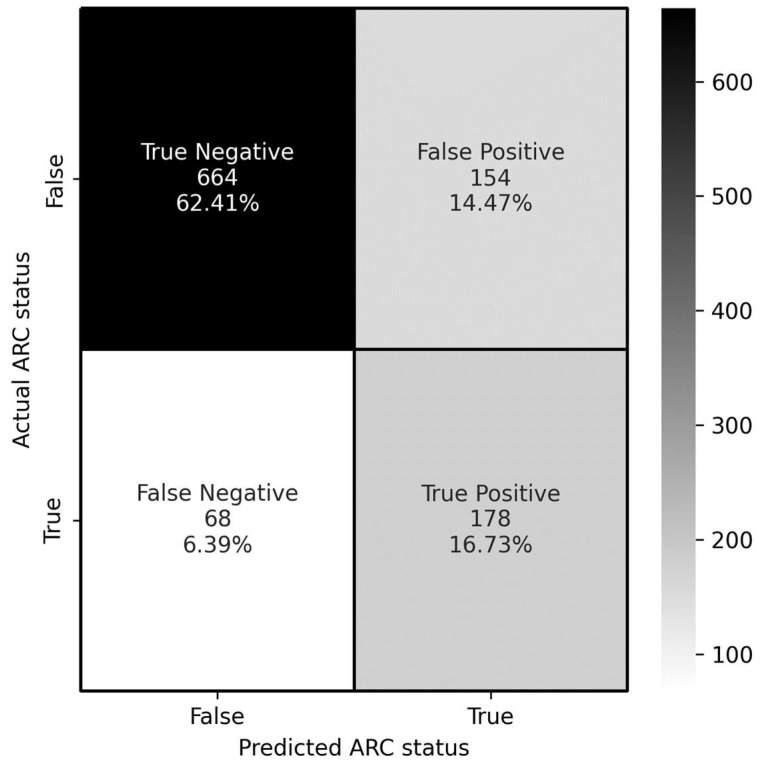
Confusion matrix with numbers and percentages of ICU days with true/false positives/negatives. ARC, augmented renal clearance.

**Figure 4:**
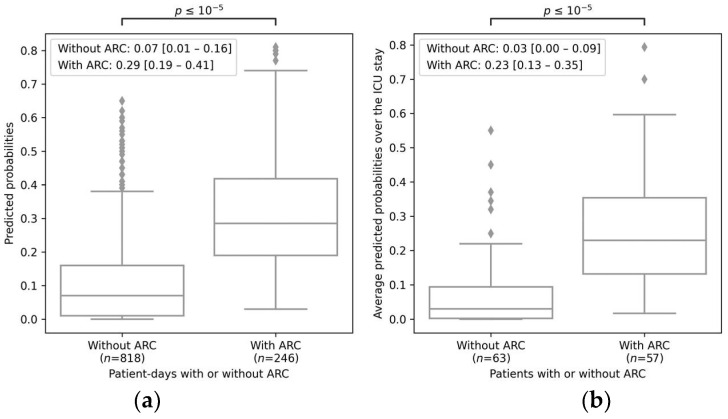
Comparison of predicted probabilities of ARC on the next ICU day between (**a**) patient-days with and without ARC and (**b**) patients with and without ARC during their ICU stay, with predicted probabilities average over their ICU stay, regardless of the presence of ARC on that day.ARC, augmented renal clearance.

The discrimination of the ARC predictor was comparable to the discrimination in the original study (AUROC: 0.86 vs. 0.89) but the calibration was slightly less (calibration slope (CS): 1.18 vs. 0.95; calibration-in-the-large (CITL): 0.14 vs. 0.12) (Figure 2). At the classification threshold of 20% (as proposed in the original study), the sensitivity, specificity, positive predictive value, negative predictive value, positive likelihood ratio, and negative likelihood ratio were 72.36%, 81.17%, 53.61%, 90.71%, 3.84, and 0.34, respectively (Figure 3), in comparison with 87.9%, 76.9%, 48.3%, 96.3%, 3.8, and 0.16, respectively, in the original study. The decision curve analysis demonstrated clinical usefulness across a broad range of classification thresholds (4.04–81.82%), similar to the original study (1–71%). The ARC predictor showed a higher area under the precision-recall curve (AUPRC) of 0.62 than the baseline AUPRC of 0.23 (Figure S2 from supplementary). The probability predictions for ARC were significantly higher in patients and patient-days with ARC compared with the probability predictions for ARC in patients and patient-days without ARC (Figure 4). On each ICU day within the first two weeks of ICU admission, predicted probabilities were significantly higher in patient-days with ARC than in patient-days without ARC (Figure S3 from supplementary).

### 2.3. ARC Predictor Feature Importance

The SCr on the previous day was the most important feature, followed by the day from admission, age, and sex, with a median permutation importance of 0.28, 0.10, 0.04, and 0.02, respectively (Figure S4 from supplementary). Since all patients were admitted due to respiratory insufficiency resulting from COVID-19, they were all without trauma or cardiac-surgery-related diagnoses on ICU admission and thus of zero permutation importance for these two features.

## 3. Discussion

In this external validation study, we found that the previously built ARC predictor had good performance in predicting the presence of ARC on the next ICU day in critically ill COVID-19 patients. Specifically, the robustness of the ARC predictor was confirmed by the comparable AUROC of the ARC predictor in this study compared to those of the original study and by the significantly higher predicted probabilities in patients and patient-days with ARC. The calibration plot expressed that the ARC predictor slightly underestimated the ARC risk in this population. Nevertheless, the decision curve analysis showed a similarly wide clinical-usefulness range, and the default classification threshold of 20% (that maximized the sensitivity and specificity in the original study) was still able to attain clinical usefulness. These results demonstrate the potential of the ARC predictor for risk stratification and drug-dose adjustment in this critically ill COVID-19 population.

Even though the discrimination was similar in this cohort to the cohort in the original study, and the clinical-usefulness range was wide, the calibration was slightly different compared with the original study. This was expected and may be explained by the significantly different patient characteristics between this critically ill COVID-19 cohort and the original ARC predictor development cohort. The development cohort included trauma and cardiac surgery patients but no COVID-19 patients. These patients have a different clinical presentation and course during their ICU stay compared with other critically ill patients [21]. Nevertheless, COVID-19 patients might experience systemic inflammatory response syndrome, which can (in-)directly overlap with the mechanism of ARC [16] and consequently increase ARC risk. In addition, a specific pathophysiological mechanism might play a role here, as viral particles have been found in the kidneys of COVID-19 patients. We also found a lower sensitivity of the ARC predictor in this cohort than in the original cohort. Both the calibration curve and the lower sensitivity indicate that the ARC predictor underestimates the risk of ARC.

Based on the permutation importance plot, the SCr of the previous day was the most important feature, which was reasonable because it directly and quickly reflected the time-variant kidney function. The second most important feature was the day from ICU admission. We found that ARC occurred relatively early after ICU admission, which is opposed to the findings of Beunders et al., who found that ARC occurred late on median (IQR) day 28 (21–42) following ICU admission during COVID-19 infections [17], but is in line with the findings of previous studies in general ICU patients (the highest ARC prevalence was observed on day 5) and critically ill COVID-19 patients (the median (IQR) first day of ARC was day 2 (3–5) of ICU stay) [1,19]. Next, age was ranked as the third most important feature, which was expected since age has consistently shown a significant inverse association with ARC in many studies [7,9,10,22,23,24,25,26,27,28,29,30,31,32,33]. Age might be more relevant in this patient population since it has been noticed that some COVID-19 variants are more prevalent in young patients [34,35]. In addition, sex was an important feature, which is not unexpected as there is a well-known association between the male sex and the occurrence of ARC [7,9,10,23,25,27,28,29,32]. Finally, the permutation importance plot revealed that these four ARC predictor features had positive permutation importance and were thus all effectively contributing to the final robust predictive performance.

Our study has many strengths. First, we reported all key measures to evaluate the model’s performance, namely the discrimination, calibration, and clinical usefulness [36]. Second, the study was reported using the Transparent Reporting of a Multivariate Prediction Model for Individual Prognosis or Diagnosis (TRIPOD) guidelines (Table S2 from supplementary) [37]. Third, this study was based on a large high-quality COVID-19 cohort without any missing value in the ARC predictor features, and thus no imputation methods were applied. Consequently, the presented results are reliable and trustworthy. Fourth, the reported permutation importance helped to understand the contribution of each ARC predictor feature in this study cohort. Finally, the higher predicted probabilities in patients and patient-days with ARC were explicitly investigated, either as a whole during the entire ICU stay, regardless of the ICU days or on each ICU day within two weeks after ICU admission.

However, our study also has several limitations. First, this is a retrospective study, so we were not able to assess whether the ARC predictor could help achieve pharmacokinetic targets, optimize renally cleared drug dosage, and/or improve patient outcomes. Second, this is a single-center Belgian study, while the optimum is to validate the ARC predictor performance in a larger multicenter international setting. Third, there might be a selection bias resulting from the exclusion criteria where patient-days with the need for temporary kidney transplant therapy (KRT), unavailable SCr on the previous day, and/or unavailable CrCl on the present ICU day were removed. However, these inclusion/exclusion criteria were necessary to ensure that only reliable CrCls were used for performance evaluation and were the same as in the original study. Finally, future studies are needed to assess whether the ARC predictor can improve the drug dosage of antibiotics and low-molecular-weight heparins.

## 4. Materials and Methods

### 4.1. Study Databases with Inclusion and Exclusion Criteria

Model validation was performed on adult COVID-19 pneumonia patients who had a positive polymerase chain reaction (PCR) for Severe Acute Respiratory Syndrome coronavirus 2 (SARS-CoV-2) on a respiratory sample and who were admitted to critical care in the University Hospitals Leuven from February 2020 to January 2021. Ethical approval was obtained from the Ethics Committee (EC) Research UZ/KU Leuven (S66365) with the study title “Machine learning tools in critically ill COVID-19 patients: external validation of the Acute Kidney Injury and Augmented Renal Clearance predictors” on 8 April 2022. The need for informed consent form was waived due to the noninterventional nature of the study. The study was conducted in compliance with the principles of the Declaration of Helsinki and its later revisions. Patients were excluded if they had end-stage kidney disease defined as chronic hemodialysis and/or kidney transplant upon ICU admission. Patient-days were excluded if they had (1) no available SCr measured on the ICU day prior to the day for which the prediction was made, (2) no measured CrCl on the ICU day for which the prediction was made, (3) KRT on the day for which the prediction was made, (4) onset of intermittent dialysis during the previous ICU days, (5) incomplete ICU day (day 0), and/or 6) KRT on the day prior to the day for which the prediction was made.

### 4.2. ARC Definition

Daily CrCl was measured for each ICU day based on the daily 24 h urine output (UO), urinary creatinine (UCr), and SCr with correction for an average body surface area: CrCl (mL/min/1.73 m^2^) = UCr (mg/dL) × 24 h UO (ml/day)/SCr (mg/dL)/1440 (min/day) × 1.73/(0.007184 × height (cm)^0.725^ × weight (kg)^0.425^). If more than one value was available on the same ICU day, the mean was applied for UCr and SCr, and the summation was applied to UO. ARC was defined as a measured CrCl larger than 130 mL/min/1.73 m^2^. Data were retrieved from the patient data management system database (Microsoft SQL Server^®^; Microsoft^®^, Redmond, Washington, WA, USA). After the application of the exclusion criteria, there were no patient-days with missing values for any ARC predictor feature.

### 4.3. ARC Predictor

The ARC predictor is a model developed by Gijsen et al. [10] to predict ARC on the next ICU day based on six routinely collected clinical variables: age, sex, day from ICU admission, SCr of the previous day, trauma-related diagnosis on ICU admission (True/False), and cardiac surgery related diagnosis on ICU admission (True/False), by using a generalized estimating equation (GEE) logistic regression with backward feature selection. The ARC predictor calculates the predicted probability with the provided six features, which can then be translated into a prediction for ARC on the next ICU day according to a prespecified classification threshold. This classification threshold is set by default at 20%, which maximized sensitivity and specificity in the original study, although the threshold can also be manually adapted.

### 4.4. Evaluation Metrics for Predictive Performance

For better interpretation and comparison with the original study, model performance was evaluated by using the same evaluation metrics: receiving operating characteristics (ROC) curve (including AUROC, sensitivity, specificity, positive predictive value, negative predictive value, positive likelihood ratio, and negative likelihood ratio), calibration plot (including CS and CITL) [38], and decision curve analysis [39]. The precision–recall (PR) curve (including AUPRC) was also examined. To further investigate the importance of each ARC predictor feature and to examine whether all features were still predictive for this study cohort, 100 repetitions of AUROC-based permutation importance were measured [40]. A boxplot was used to compare the predicted probabilities between (i) the patient-days with and without ARC during the entire ICU stay and on each ICU day within two weeks after ICU admission; (ii) the patients with and without ARC where the predicted probabilities were averaged over their ICU stay, regardless of the presence of ARC on that day. Additionally, the percentage of ARC days and the number of ARC days in ARC patients were investigated and visualized with a boxplot.

### 4.5. Descriptive Analyses and Software Used

All analyses were performed in Python 3.7.4 (Python Software Foundation, http://www.python.org (accessed on 19 May 2022)) with SciPy version 1.7.3 (SciPy.org (accessed on 19 May 2022)) and Scikit-learn library 1.0.2 (scikit-learn.org (accessed on 19 May 2022)). Descriptive statistics were used to describe the study population, with continuous data presented as medians, and IQR and categorical data expressed as counts and percentages (%). To evaluate the statistical significance of differences, a GEE model was used with the patient identification number as the grouping variable. A two-tailed P-value less than or equal to 0.05 was considered statistically significant.

## 5. Conclusions

In conclusion, we demonstrated the robustness of the ARC predictor to predict ARC on the next ICU day in critically ill COVID-19 patients, based on six routinely collected clinical variables in the ICU. Despite the promising performance, these findings should be prospectively validated in independent patient populations before the ARC predictor can be implemented for risk stratification or used to inform optimized dosing strategies in routine clinical ICU practice.

## Figures and Tables

**Table 1:** Patient characteristics and clinical outcomes (per patient).

Variables	All Patients (*n* = 120)	ARC (*n* = 57, 47.50%)	Not ARC (*n* = 63, 52.50%)	*p*-Value
Age, years, median (IQR)	67 (59–75)	61 (57–67)	73 (65–78)	<0.01
Sex male, number (%)	87 (72.5)	40 (70.2)	47 (74.6)	0.59
Height, m, median (IQR)	1.7 (1.6–1.8)	1.8 (1.7–1.8)	1.7 (1.6–1.8)	0.03
Weight, kg, median (IQR)	85.0 (71.5–104.0)	86.0 (70.0–104.0)	85.0 (73.5–101.5)	0.63
BMI, median (IQR)	28.7 (25.8–33.1)	28.1 (26.1–32.8)	29.0 (25.8–34.3)	0.71
Baseline serum creatinine, mg/dL, median (IQR)	0.9 (0.8–1.0)	0.9 (0.7–0.9)	0.9 (0.9–1.1)	<0.01
APACHE II score, median (IQR)	19 (15–25)	18 (13–23)	19 (17–27)	<0.01
Day from ICU admission, day, median (IQR)	6.0 (3.5–10.0)	6.5 (4.0–10.0)	6.0 (3.2–10.5)	0.73
Creatinine clearance, mL/min/1.73 m^2^, median (IQR)	91.3 (54.7–132.5)	133.8 (106.4–165.0)	55.9 (27.8–81.9)	<0.01
Length of stay in ICU, days, median (IQR)	14 (9–24)	15 (8–24)	14 (10–24)	0.11

BMI, body mass index; APACHE II score, Acute Physiology and Chronic Health Evaluation II score; IQR, interquartile range.

## Data Availability

The datasets generated and/or analyzed during the current study are not publicly available due to no prior agreement with the ethical committee but are available from the corresponding author on reasonable request.

## Supplementary Materials

The following supporting information can be downloaded at: https://www.mdpi.com/article/10.3390/antibiotics12040698/s1, Figure S1: Percentage of ARC days (left) and number of days with ARC (right) in ARC patients; ARC, augmented renal clearance; Figure S2: Precision–recall curve. AUPRC, area under the precision-recall curve. Baseline, the number of positive cases (patient-days with the presence of augmented renal clearance) over the total number of patient-days; Figure S3: Comparison of predicted probabilities of ARC on each ICU day between patient-days with and without ARC, within the first two weeks of ICU admission. The black and grey numbers above the figure indicate the numbers of patient-days with and without ARC on each ICU day, respectively. ARC, augmented renal clearance; Figure S4: Boxplots of permutation importance of all augmented renal clearance predictor features with 100 repetitions. AUROC, area under the receiver operating characteristics curve; Table S1: Patient characteristics and clinical outcomes (per patient-day); Table S2: Transparent reporting of a multivariable prediction model for individual prognosis or diagnosis (TRIPOD) statement.
