# Supplementary material for: External Validation of the Augmented Renal Clearance Predictor in Critically Ill COVID-19 Patients
Source: Antibiotics (Basel). 2023 Apr 3;12(4):698. doi: 10.3390/antibiotics12040698 (PMC10135364; doi:10.3390/antibiotics12040698)
Supplement: Supplementary file 1 [file antibiotics-12-00698-s001.zip › antibiotics-2272119-supplementary.pdf]

Supplementary Tables

Table S1 Patient characteristics and clinical outcomes (per patient-day)

Variables	All patient-days (n=1064)	ARC (n=246, 23.12%)	Not ARC (n=818, 76.88%)	p-value
**Age, years, median (IQR)**	65 (60 – 72)	60 (52 – 65)	70 (60 – 73)	0.03
**Gender male, number (%)**	805 (75.7)	173 (70.3)	632 (77.3)	0.35
**Height, m, median (IQR)**	1.8 (1.6 – 1.8)	1.8 (1.7 – 1.8)	1.7 (1.6 – 1.8)	0.19
**Weight, kg, median (IQR)**	85.0 (75.0 – 104.0)	86.0 (70.0 – 105.0)	85.0 (76.5 – 103.0)	0.73
**BMI, median (IQR)**	28.7 (26.2 – 32.1)	28.4 (26.3 – 32.1)	28.7 (26.2 – 31.9)	0.19
**Baseline serum creatinine, mg/dl, median (IQR)**	0.9 (0.8 – 0.9)	0.9 (0.7 – 0.9)	0.9 (0.8 – 1.0)	<0.01
**APACHE II score, median (IQR)**	19 (15 – 25)	17 (14 – 22)	19 (16 – 25)	<0.01
**Day from ICU admission, day, median (IQR)**	9.0 (4.0 – 18.0)	8.0 (4.0 – 13.0)	10.0 (5.0 – 20.0)	<0.01
**Creatinine clearance, ml/min/1.73m^2, median (IQR)**	91.9 (60.4 – 127.9)	152.7 (138.3 – 175.6)	74.8 (52.3 – 102.2)	<0.01
**Length of stay in ICU, days, median (IQR)**	22 (13 – 38)	18 (11 – 23)	24 (15 – 44)	<0.01

Statistically significant difference was examined by using univariable generalized estimating equation (GEE) model, with ICU admission number as clustering variable, and days with ARC as reference group. BMI, body mass index; APACHE II score, Acute Physiology and Chronic Health Evaluation II score; IQR, interquartile range

Table S2 Transparent reporting of a multivariable prediction model for individual prognosis or diagnosis (TRIPOD) statement

Section/Topic	Item		Checklist Item	Location
**Title and abstract**				
Title	1	D;V	Identify the study as developing and/or validating a multivariable prediction model, the target population, and the outcome to be predicted.	Title
Abstract	2	D;V	Provide a summary of objectives, study design, setting, participants, sample size, predictors, outcome, statistical analysis, results, and conclusions.	Abstract
**Introduction**				
Background and objectives	3a	D;V	Explain the medical context (including whether diagnostic or prognostic) and rationale for developing or validating the multivariable prediction model, including references to existing models.	Background
	3b	D;V	Specify the objectives, including whether the study describes the development or validation of the model or both.	Background
**Methods**				
Source of data	4a	D;V	Describe the study design or source of data (e.g., randomized trial, cohort, or registry data), separately for the development and validation data sets, if applicable.	Methods: Study databases with inclusion and exclusion criteria
	4b	D;V	Specify the key study dates, including start of accrual; end of accrual; and, if applicable, end of follow-up.	Methods: Study databases with inclusion and exclusion criteria
Participants	5a	D;V	Specify key elements of the study setting (e.g., primary care, secondary care, general population) including number and location of centres.	Methods: Study databases with inclusion and exclusion criteria
	5b	D;V	Describe eligibility criteria for participants.	Methods: Study databases with inclusion and exclusion criteria
	5c	D;V	Give details of treatments received, if relevant.	N.A.

Outcome	6a	D;V	Clearly define the outcome that is predicted by the prediction model, including how and when assessed.	Methods: ARC definition, and ARC predictor
	6b	D;V	Report any actions to blind assessment of the outcome to be predicted.	N.A.
Predictors	7a	D;V	Clearly define all predictors used in developing or validating the multivariable prediction model, including how and when they were measured.	Methods: ARC predictor
	7b	D;V	Report any actions to blind assessment of predictors for the outcome and other predictors.	N.A.
Sample size	8	D;V	Explain how the study size was arrived at.	Results: Study cohort
Missing data	9	D;V	Describe how missing data were handled (e.g., complete-case analysis, single imputation, multiple imputation) with details of any imputation method.	ARC features have no missing values
Statistical analysis methods	10a	D	Describe how predictors were handled in the analyses.	Methods: ARC predictor
	10b	D	Specify type of model, all model-building procedures (including any predictor selection), and method for internal validation.	Methods: ARC predictor
	10c	V	For validation, describe how the predictions were calculated.	Methods: ARC predictor
	10d	D;V	Specify all measures used to assess model performance and, if relevant, to compare multiple models.	Methods: Evaluation metrics for predictive performance
	10e	V	Describe any model updating (e.g., recalibration) arising from the validation, if done.	N.A.
Risk groups	11	D;V	Provide details on how risk groups were created, if done.	Methods: ARC definition
Development vs. validation	12	V	For validation, identify any differences from the development data in setting, eligibility criteria, outcome, and predictors.	Methods: Study databases with inclusion and exclusion criteria, and ARC definition, and ARC predictor

**Results**				
Participants	13a	D;V	Describe the flow of participants through the study, including the number of participants with and without the outcome and, if applicable, a summary of the follow-up time. A diagram may be helpful.	Results: Study cohort
	13b	D;V	Describe the characteristics of the participants (basic demographics, clinical features, available predictors), including the number of participants with missing data for predictors and outcome.	Results: Study cohort
	13c	V	For validation, show a comparison with the development data of the distribution of important variables (demographics, predictors and outcome).	Results: Study cohort
Model development	14a	D	Specify the number of participants and outcome events in each analysis.	N.A.
	14b	D	If done, report the unadjusted association between each candidate predictor and outcome.	N.A.
Model specification	15a	D	Present the full prediction model to allow predictions for individuals (i.e., all regression coefficients, and model intercept or baseline survival at a given time point).	N.A.
	15b	D	Explain how to the use the prediction model.	N.A.
Model performance	16	D;V	Report performance measures (with CIs) for the prediction model.	Results: ARC predictor external validation performance
Model-updating	17	V	If done, report the results from any model updating (i.e., model specification, model performance).	N.A.
**Discussion**				
Limitations	18	D;V	Discuss any limitations of the study (such as nonrepresentative sample, few events per predictor, missing data).	Discussion: the last paragraph
Interpretation	19a	V	For validation, discuss the results with reference to performance in the development data, and any other validation data.	Discussion: the second paragraph
	19b	D;V	Give an overall interpretation of the results, considering objectives, limitations, results from similar studies, and other relevant evidence.	Discussion: the third paragraph
Implications	20	D;V	Discuss the potential clinical use of the model and implications for future research.	Discussion: the first paragraph
**Other information**				

Supplementary information	21	D;V	Provide information about the availability of supplementary resources, such as study protocol, Web calculator, and data sets.	Supplementary digital content
Funding	22	D;V	Give the source of funding and the role of the funders for the present study.	Title page

*Items relevant only to the development of a prediction model are denoted by D, items relating solely to a validation of a prediction model are denoted by V, and items relating to both are denoted D;V. N.A., not applicable.

Supplementary Figures

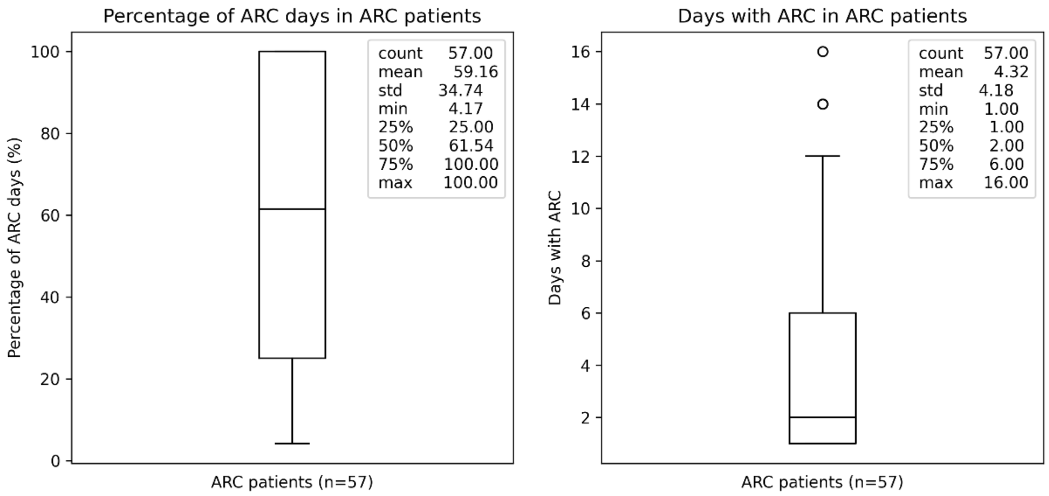

Figure S1 Percentage of ARC days (left) and number of days with ARC (right) in ARC patients; ARC, augmented renal clearance

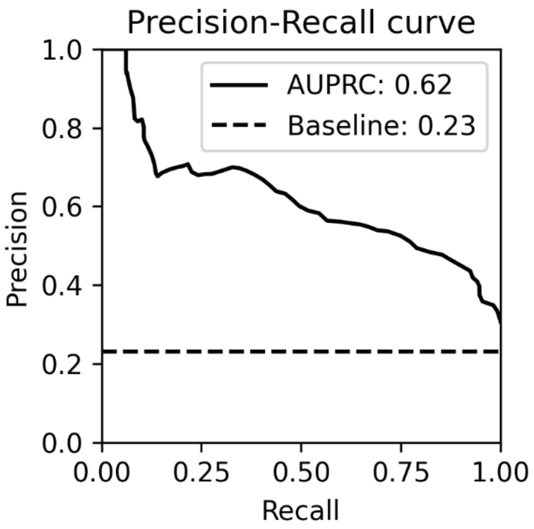

Figure S2 Precision recall curve. AUPRC, area under the precision recall curve. Baseline, the number of positive cases (patient-days with presence of augmented renal clearance) over the total number of patient-days

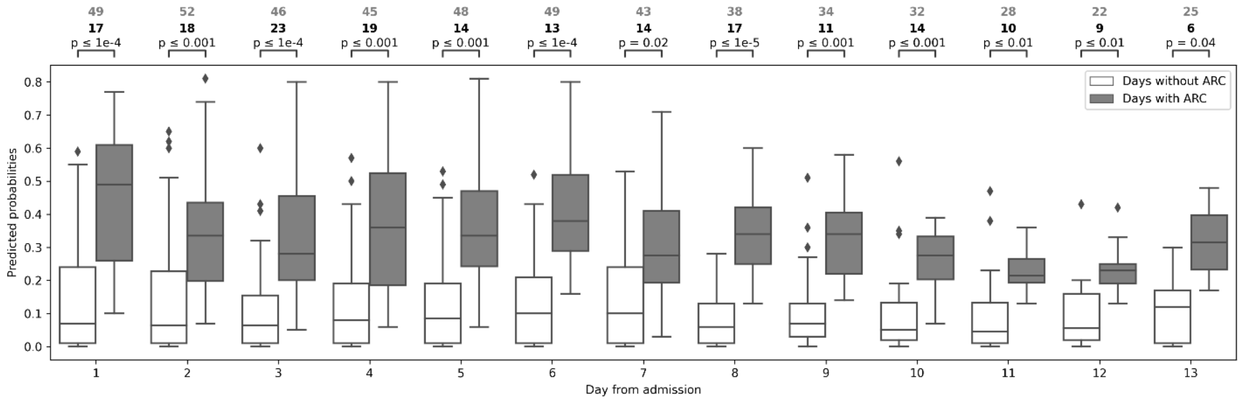

Figure S3 Comparison of predicted probabilities of ARC on each ICU day between patient-days with and without ARC, within the first two weeks of ICU admission. The black and grey numbers above the figure indicated the numbers of patient-days with and without ARC on each ICU day. ARC, augmented renal clearance

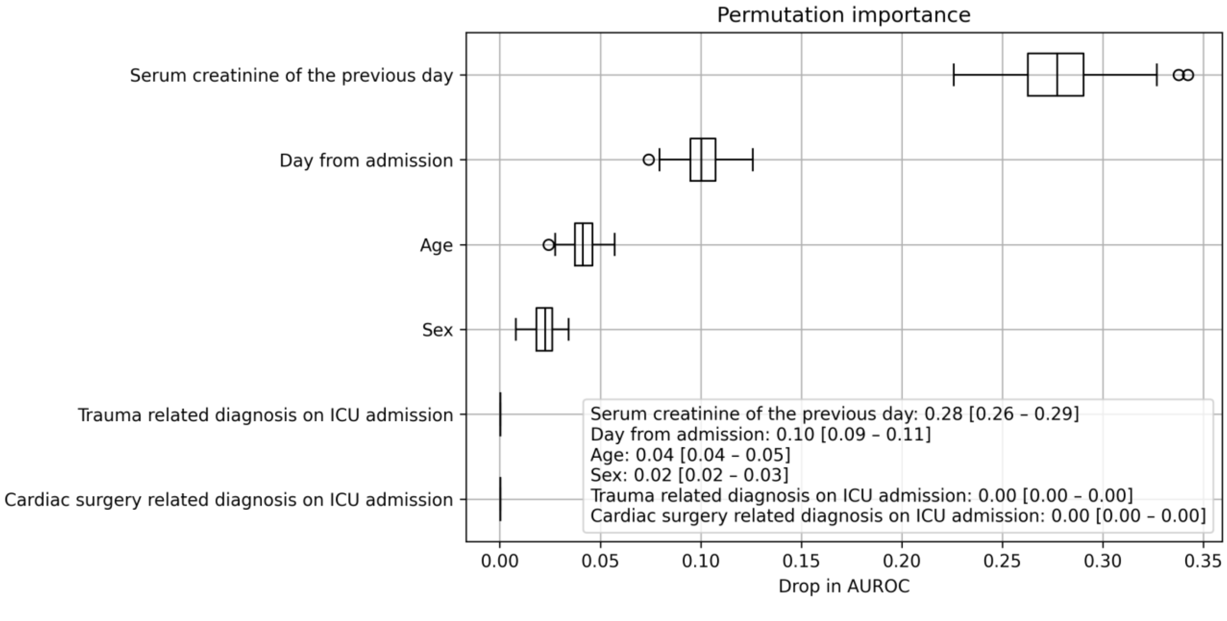

Figure S4 Boxplots of permutation importance of all augmented renal clearance predictor features with 100 repetitions. AUROC, area under the receiver operating characteristics curve
